# Foreign Medical Literature

**Published:** 1819-04-01

**Authors:** 


### THIRD DEPARTMENT.

### Foreign Medical Literature.

### I.

*On the Distinction between Active and Passive Aneurism of the Heart, with Cases and Practical Remarks.* By M. Rostan.

A blind submission to the authority of great names, is, without doubt, the surest way to perpetuate the infancy of science. *Are those causes which are said to induce active or passive aneurism of the heart and the symptoms which attend them, ascertained by actual observation?*

"Active aneurism, says M. Corvisart, results from sanguine temperament, robustness, youth, hard labour, violent exertions, long journeys on foot or on horseback, venereal excesses, stimulating food, inebriety, strong exertions of the voice, violent emotions of the mind, &c." "The accompanying symptoms, according to the same author, are, redness of the face, tumultuous action of the heart, visible to the eye, or tangible by the hand; strength, hardness, and vibration in the pulse, throbbing of the carotid arteries, &c."

To passive aneurism he assigns causes and symptoms of an opposite nature. Let us examine these by the test of experience. The following cases occurred among people who were arrived at an advanced age, and who lived under the influence of debilitating causes, as innutritious diet; ennui; habitual inactivity; absence from their families; the depressing passions, &c.; and who were, moreover, affected with chronic diseases:—yet nothing was so rare as *passive* aneurism among them. M. Corvisart himself, on the contrary, relates the case of a young mantua maker, 24 years of age, of a feeble constitution, with small, weak pulse, who, nevertheless, presented on dissection, a well marked *active* aneurism.

Case i. Frances Dumay, 65 years of age, of a weak and worn out constitution, had experienced, during sixteen months, violent palpitation of the heart, and difficulty of breathing, succeeding the healing up of an old ulcer. She applied for relief at the Salpetriere, on the 17th of April, 1817. Her face was tumid and red; the eye-lids swelled; the upper extremities covered with scorbutic eruptions. The respiration was short; but the dyspnœa generally came on at three o'clock in the morning, and disappeared in the course of the day. At this time she can only breathe in the erect position; the palpitation is sufficiently evident; the pulse pretty regular, but weak; the appetite considerable. Several blisters and venesection produced no good effect. On the night of the 20th the patient experienced several severe attacks of dyspnœa, which had been mitigated by an antispasmodic mixture, with tincture of digitalis. 23d. The lower extremities are œdematous; in other respects the same symptoms continue. 27th. In the morning, the patient expired.

*Sectio Cadaveris.* All the extremitie sœdematous. On the *right* side of the thorax, adhesions between the lung and costal pleura; lung itself gorged. In the *left* side some effusion; lung itself hepatized; the membrane lining the bronchia red; the air passages clogged with mucus. Heart enormously enlarged; hard; the parietes of the left ventricle fifteen lines in thickness; the cavity very small. The aorta was ossified; its internal surface red; and its calibre alternately dilated and contracted.

*Remarks.* It may be observed that the pulse, in this case, preserved its regularity of rythm to the last; but in a majority of instances, it is irregular, unequal, and intermittent. It has been remarked by Landré-Beauvais, in his excellent work on Semeiology, that in old people it is not uncommon to meet with these irregularities in the pulse, while they appear otherwise in good health. These phenomena, however, are not the less indicative of cardiac lesion, though in a slighter degree.

Case ii. A woman, 73 years of age, of a lymphatic temperament, who had experienced, for twenty-eight years past, a difficulty of breathing, especially in the winter, and during the night, entered the Saltpetriere on the 5th of March, 1817. Her face was pale, livid, and somewhat puffed; her lips of violet hue; her skin shining and scaly. During the last eight months she was harrassed with cough and dyspnœa. She now kept in the perpendicular position for some days, in order to breathe easier; afterwards she was able to lie down on the *right* side. This side of the thorax sounded badly on percussion, especially towards the inferior ribs. The sputum was opake, homogeneous, and of a yellowish green colour. The pulse was more frequent and strong than natural, with intermissions at irregular periods; the pulsations of the heart more sensible to the hand than in a state of health; no appetite; general debility. *March* 11*th*. Enlargement of the right side of the chest; swelling of the upper and lower extremities of the same side. By the 2d of April, these swellings had disappeared; the patient was a little delirious, and lay on her back. Died in the night of the 5th of April.

*Dissection.* The *right* lung sound, but that side of the chest greatly encroached on by an *unusual elevation of the Liver*. The inferior lobe of the *left* lung hepatized; the pleura of this side coated with a soft, yellowish, false membrane; bronchi red. Heart greatly enlarged; both ventricles *actively* dilated, and of great thickness; ossification of the aortic valves, and of several points of the aorta itself.

*Remarks.* The dull sound which the *right* side of the chest emitted, was probably owing to the remarkable elevation of the liver; and it would appear that the hepatization of the inferior lobe of the left lung had only occurred in the latter few days of this woman's life, during which time we did not use percussion.

We may here also observe that the greater number of aneurismatic patients die of *inflammation* of some organ; generally of the lungs or intestines. M. Bayle often found the coats of the stomach red:—all indeed shew marks of congestion in some viscus or other. The stagnation of blood resulting from the embarrassed state of the circulating organ explains this circumstance.

Case iii. Mary Lacour, 82 years of age, entered the Saltpetriere the 24th March, 1817, in a state of general debility, occasioned, it was supposed, from old age alone, and that to such a degree, that she was unable to move herself. Her face was bloated and of a violet colour, in some points, as over the cheek bones, nose, and chin. The circumference of the nostrils, mouth, and eyes, was of an earthy tint. The faculties of perception and the organs of sense were almost completely torpid; the respiration was rattling, espscially when asleep; occasional cough without expectoration; *pulse small, soft, and irregular.*

This state of debility advanced rapidly; the integuments over the sacrum mortified; and she expired in the night of the 5th of April.

*Dissection.* Considerable effusion in both sides of the chest; lungs sound; left ventricle of the heart very thick and dense; its cavity almost obliterated. Ossifications the size of a pistachio nut in the margins of the aortic, and auriculo-ventricular openings. Some water in the brain.

Case iv. On the 25th of March 1817, a woman, 75 years of age, died in one of the wards of the Saltpetriere, where she had been treated for disease of the heart, accompanied by symptoms of peripneumony. The minutes of her illness represented the pulse as *small, frequent, and easily compressible.*

*Dissection.* Right side of the chest shewed strong, and long standing adhesions between the costal and pulmonary pleura. A soft and recent exudation covered the diaphragm of this side ; the two inferior lobes of the lungs hepatized. Bronchi red. Heart enormously enlarged. The parietes of the ventricles, particularly of the left ventricle, more than an inch in thickness. Considerable ossification of the aorta.

Case v. Catherine Malhere, 75 years of age, subject for fifty years, to dyspnœa and flatulency, entered the Saltpetriere on the 5th of July, in a state of inexpressible anxiety and difficulty of breathing, which had continued, more or less violent, for ten days past. She spent the night in the erect posture. Next morning we found the countenance much altered ; the respiration panting ; the suffocation imminent ; the cough frequent ; the expectoration difficult ; the sputum opake. The patient tossed about her limbs ; had frequent eructations ; *pulse small, and irregular ;* strong palpitations ; lower extremities oedematous. In about fourteen days there was a considerable mitigation of the symptoms ; but indications of enteritis now appeared, with low fever, and she died on the 30th of July.

*Dissection.* Entire adhesion between the pulmonary and costal pleura of the right side ; lung of the same side of unnaturally firm consistence, but still crepitous. In the left cavity of the pleura, much effusion. Pericardium adherent to the heart ; the latter organ as large as a child's head, of a year old ; the parietes of all its chambers thickened ; considerable ossifications in the aorta. In the abdomen the most intense inflammation existed throughout the whole intestinal canal.

Case vi. A woman, 67 years of age, of nervous temperament, and very irritable, applied every year, for a considerable time past, to the Saltpetriere, on account of dyspnœa and palpitation which disappeared in summer, but always recurred in winter. In December 1815, this woman was in M. Pinel's wards, and derived temporary relief from æther, squills, &c. In November, 1816, she came into my wards, when I found the following symptoms. Considerable emaciation ; dyspnœa ; orthopnœa ; frequent and distressing cough, with mucous, glary expectoration ; pulse small, unequal, intermittent ; no appetite ; constipation obstinate.

These symptoms continued with more or less intensity, when she was overtaken with such debility as forced her to keep her bed. Anasarca now supervened, aud death closed the scene.

*Dissection.* General infiltration ; a pint of cloudy serum in the right thoracic cavity ; pleura thickened, and covered with a false membrane ; lung splenified ; heart greatly enlarged ; and covered with whitish patches ; walls of the right ventricle very much thickened; aorta diseased. *Abdomen.* The mucous membrane of the intestines of a violet colour.

We could multiply cases of this kind to any extent from the practice of the Saltpetriere, but the reader will not consider it necessary. It may be proper to state, however, that of 81 cases entered on the books as supposed dangerous affections of the heart, in the months of March and April, 1817, thirty-six have died; of which number, twenty-six were found to be aneurisms of this organ; and of this last number, twenty-two were *active* and only four *passive*.

From these facts it results as a fair conclusion, that *active* aneurism of the heart is of very frequent occurrence in old people, and is principally owing to ossification of the aorta. Hence, too, it follows that youth, adult age, sanguine temperament, and various other supposed exciting causes, are not always necessary to the production of active aneurism. The pulse, far from being always *strong, hard, and vibrant*, is often *small, contracted,* [serré] *soft*, or even scarcely perceptible. These characters are so much the more strongly marked as the obstruction to the circulation is greater, in consequence of which obstruction, the heart has acquired a larger volume.

Aneurism of the heart is so frequently met with in old people, that very few die at an advanced age, without traces of it, more or less distinctly marked; a fact which is well ascertained in the Saltpetriere, on account of the great number of dissections of very old patients there.

Corvisart attributes the formation of cardiac aneurisms "to obstruction to the free course of the blood, from any cause, physical or moral." Experience has shewn that the accumulation of phosphate of lime on the coats of the great arteries, (the inevitable consequence of age) is the common cause of aneurism of the heart in old people. We found, about four years ago, a most remarkable enlargement of the heart, without any ossification of the valves, &c. and we had laid the subject aside, with the belief that the obstruction existed at some distance from the centre of the circulation. One of our pupils, in fact, prosecuted the dissection, and discovered that the calibre of the aorta was almost obliterated from its entrance into the abdomen till its division into the iliacs. A similar case presented itself to our observation within these few days. In rickety subjects, also, where there is a bad conformation of the chest, and where the lungs are confined in a narrow space, we have generally found *active* aneurism of the right ventricle, whatever was the age or the constitution of the patient. M. Bayle has made a similar observation.

NOTE BY THE ENGLISH EDITORS.

It would appear that M. Rostan has overlooked other causes of *active* aneurism of the heart, than obstruction to the circulation. We have dissected several bodies where the heart was actively enlarged, from successive attacks of inflammation in the organ itself, particularly from translated gout and rheumatism, and where no visible cause of obstructed circulation existed. We quite agree with M. Rostan, however, that *active* is infinitely more common than *passive* aneurism, and that our curative views should generally hinge on this consideration.

---

### II.

### *Semeiological Observations.* Partly from the French.

Section I. *Posture.*—The attitude which a sick man assumes, during sleep, is often indicative of the state of certain functions, and is not to be overlooked by the attentive physician.

1. In health, the limbs are all in a state of demi-flexion and relaxation; but this must be carefully distinguished from a somewhat similar appearance which takes place in disease, from a total loss of muscular power, and which is indicative of the greatest danger.

2. In inflammatory diseases, in the greater number of inflammations, and at the approach of eruptions, the patient is so tormented with heat and anxiety, that he is *constantly changing his position.* In the worst species of fevers, on the other hand, he lies on his back, in a state of *supination,* which is at once the sign and the effect of prostration of strength. The muscles have so little power that the whole body gravitates as it were towards the earth, from whatever position it is placed in—hence the appearance of the patient descending towards the foot of the bed, because it is lower than the head.

3. If the patient, while in a posture of supination, has the lower extremities thrown asunder, and the arms, feet, neck, and breast uncovered, although these parts are cool, the danger is great. The same applies to the half open state of the eyes and mouth, with the head reclining backwards.

4. To find a patient lying on his face is a bad sign, as it indicates approaching delirium, or severe abdominal pains. It is a good sign when the patient tries to find an easy position, by turning himself from side to side; especially when he can lie a good while in the position so chosen, in a state of demi-flexion.

5. In peritonitis, the easiest position is on the back; but when one of the abdominal viscera is affected, the patient can more frequently lie on *that* than on the sound side, on account of the tension produced by the latter position. To this, however, there are many exceptions.

6. In *pleurisy,* the patient almost always lies on the sound side; when suppuration takes place, it is just the reverse.

7. In *peripneumony,* the danger is great, when the dyspnœa obliges the patient to sit up.

8. In *phthisis*, there is nothing very certain to be learnt from the decubitus. In general, however, the patient lies on the diseased side.

SECTION II. *Indications drawn from the Size.*

1. In inflammatory fever, the face, and even the whole cutaneous surface is augmented in volume. The same takes place in eruptive diseases. The augmentation commences generally in the face, and extends successively to the trunk and members. If it do not observe this order, there is danger of metastasis to some organ.

2. Chronic inflammation of an internal viscus is almost always accompanied by increase of size in the organ affected.

3. In general, it is a good sign when the size of the body does not alter much during disease; but if the disease, when violent, and one that is usually accompanied by emaciation, does not produce this effect, the danger is greater; as in low nervous fevers, where the size remains nearly stationary, although no nourishment is taken in.

4. An external swelling is, in general, favourable in acute diseases, as it relieves internal organs, not only of an overplus of blood, but determines irritation from thence to the exterior. Thus in quinsy, the more the neck is swelled, the less danger there is in the throat.

5. In convalescence, it is a good sign to see the patient return to his ordinary size, or even a little beyond that. Men who become corpulent at an *early* age, seldom live to an *advanced* one.

6. It is a good sign when maniacs and melancholics regain their size, in proportion as the mental alienation subsides. But if the body becomes corpulent while the mental disease remains stationary, there is little hope of cure.

7. Every one knows that augmentation of size from œdematous effusion, is an unfavourable symptom. A slight œdema, however, of the lower extremities, after acute diseases, is little to be feared, since the previous disease is often relieved by this effusion, which soon afterwards disappears.

8. Œdema of the hands, feet, eye-lids, or face, supervening on chronic diseases is a very bad sign.

9. In hydrothorax, the external œdema of the face, hands, &c. is generally more developed on the side that corresponds with the internal effusion, where this is confined to one side.

10. In old people a progressive diminution in size, however slow, without any apparent disease, evinces dissolution at no great distance.

11. In acute diseases, the emaciation does not proceed rapidly during the early periods; its manifests itself most sensibly in the second stage, especially about the turn of the disease.

12. The sooner the emaciation begins, the shorter the disease, and *vice versa*. This was observed by Hippocrates, who says, "if in acute fever the body suffers no diminution, the disease will be tedious." (*Aphor.*)

13. It does not appear correct to attribute the great emaciation of febrile patients to the dissipation of the juices by the fever, while no reparation is made by the ingesta. This dissipation is, in a great measure, ideal. In fact, during the first stage of fever, all the excretories are closed, and the excretions and secretions almost totally suspended. In *this* period, the emaciation is *partial*; that is, it is caused by an unequal distribution of the fluids, which gravitate, as it were, towards the points of lesion, and desert the other parts of the body. In acute diseases, as was indeed observed by Hippocrates, a sudden and rapid emaciation is a dangerous symptom; and seems to be owing to the too great activity of the absorbents, which hurry into the circulation the richest parts of the body, while the torpor of the secretory vessels prevents a sufficient outlet; hence the sanguiferous system becomes surcharged, and inflammation or congestion of internal organs is the consequence.

14. If, at the close of an acute disease, no emaciation has taken place, there is every chance of a relapse.

15. In pregnant women, the afflux of fluids towards the uterus causes an emaciation of the other parts. This is not dangerous. If, however, an extreme and sudden emaciation takes place, without apparent cause, there is danger of a laborious accouchement, or even abortion.

16. In most chronic visceral affections, there is emaciation of the other parts of the body. Thus, in chronic engorgements, in hydrothorax, ascites, encysted dropsies, &c. we constantly remark a wasting of the members, followed, at a subsequent period, by œdema of the same.

### Section III. *Colour.*

During health, many circumstances affect the colour of the skin; thus the cold bath, fear, hæmorrhages, vomiting, diarrhœa, &c. render it pale. Prisoners, and those who do not go much into the open air, are also of a pallid aspect.

The skin is red, injected, dry, &c. in inflammatory diseases; while in hepatic and gastric complaints, it assumes all the tints between deep yellow and a greenish pallor. Scirrhous affections of the stomach impress a peculiar complexion and physiognomy on the countenance—a kind of pale yellow with an earthy hue; the features being all sharpened, and something painful even to behold in the expression of the face.

Organic diseases of the heart give quite a different appearance to the skin, which is generally tinged with a violet shade, especially about the lips. Cancerous affections of the womb impress a modification of those resulting from gastric scirrhus. The tints are not so yellow or sallow as in the latter, but much more exanguious. Every one knows what important information is to be drawn from the colour and appearances of the tongue, the urine, and the fæces. And it is earnestly hoped, that the foregoing sketch may excite the curiosity of the young physician to a branch of diagnosis and prognosis that is too little studied.

